# Past environments modulate response to fluctuating temperatures in a marine fish species, *Sebastes fasciatus*

**DOI:** 10.1242/jeb.251288

**Published:** 2025-11-04

**Authors:** Christelle Leung, Joëlle J. Guitard, Caroline Senay, Audrey Bourret, Denis Chabot, Geneviève J. Parent

**Affiliations:** ^1^ Laboratory of Genomics, Maurice Lamontagne Institute, Fisheries and Oceans Canada, Mont-Joli, QC, Canada, G5H 3Z4; ^2^ Institut des sciences de la mer, Université du Québec à Rimouski, Rimouski, QC, Canada, G5L 2Z9; ^3^ Maurice Lamontagne Institute, Fisheries and Oceans Canada, Mont-Joli, QC, Canada, G5H 3Z4

**Keywords:** Phenotypic plasticity, Reaction norms, Gene expression, Global warming, Acclimation

## Abstract

Predicted ocean warming will impact the survival and structure of various marine organisms, in particular ectotherms. Phenotypic plasticity enables species to cope with environmental changes, providing a vital buffer for evolutionary adaptations. Yet, the dynamics and molecular mechanisms underpinning these plastic responses remain largely unexplored. Here, we assessed the impact of temperature acclimation on the capacity of organisms for thermal plasticity. We conducted a genome-wide transcriptomic analysis on the Acadian redfish, *Sebastes fasciatus*, exposed to four temperatures (2.5, 5.0, 7.5 and 10.0°C) over a long-term period (up to 10 months) followed on some individuals by a short-term temperature change (+2.5°C or −2.5°C for 24 h), simulating natural temperature variation the species could encounter. Our results showed a dynamic transcriptional response to temperature involving various gene functions. The rapid response to temperature shifts, coupled with the sustained expression of specific genes over an extended period, highlighted the capacity of this species for plasticity in response to temperature changes. We also detected a significant effect of the interaction between the long- and short-term temperature exposures on gene expression, highlighting the influence of the past environment on the response to short-term temperature changes. Specifically, fish acclimated to higher temperatures demonstrated an increased stress-related response to environmental fluctuations, as evidenced by both the shape of their reaction norms and the involvement of stress-related gene functions. This result suggests that temperature conditions predicted for the near future in the Northwest Atlantic will trigger reduced adaptive plasticity to environmental fluctuations, highlighting the vulnerability of this species to ocean warming.

## Teaser

**Summary:** Thermal plasticity in Acadian redfish is shaped by past temperature exposure, revealing vulnerability to future ocean warming through stress-related transcriptomic responses.

## INTRODUCTION

Under all climate change scenarios studied by the Intergovernmental Panel on Climate Change (IPCC), temperature is projected to rise, in terms of both average and intensity of fluctuations (Diffenbaugh et al., 2017; Hoegh-Guldberg et al., 2018; IPCC, 2022; Oliver et al., 2018; Seneviratne et al., 2021). This phenomenon also occurs in the oceans, driving major shifts in marine biodiversity, mainly impacting the distribution of marine species (Perry et al., 2005) and diminishing the performance, fitness and survival of organisms (Poloczanska et al., 2013; Ramírez et al., 2017; Smale et al., 2019; Smith et al., 2023). The adverse effect of ocean warming may be particularly severe for ectothermic organisms, as they lack effective physiological mechanisms to control body temperature and often operate near their thermal limits (Alfonso et al., 2021; Angilletta, 2009; Pinsky et al., 2019). Anticipating how marine communities may change under future conditions therefore requires a better understanding of the different mechanisms underlying the ability of organisms to respond effectively to environmental changes, a critical aspect for their persistence in natural environments.

Phenotypic plasticity is the capacity of a given genotype to produce different phenotypes in response to environmental conditions (Scheiner, 1993). If plasticity is adaptive, it involves the development of a phenotype suited to future conditions, thereby increasing fitness under fluctuating and heterogeneous environments (Botero et al., 2015; West-Eberhard, 2003). As such, plasticity can serve as a powerful and effective mechanism to cope with short-term environmental changes, provided that these changes are sufficiently predictable. In this context, the reliability of environmental cues becomes a key determinant of the adaptiveness of plastic responses (Bonamour et al., 2019; Gavrilets and Scheiner, 1993; Leung et al., 2020; Reed et al., 2010; Scheiner, 2013; Scheiner and Holt, 2012; Tufto, 2015). Understanding an organism's capacity for plasticity therefore requires a thorough examination of the temporal dynamics of environmental conditions. Past environments, in particular, can influence the initial phenotype, shaping the extent of phenotypic change required to produce a better-adapted phenotype in a new environment (Fey et al., 2019; Kremer et al., 2018). Moreover, past environments can provide valuable cues for phenotypic adjustment, helping recalibrate the triggers for plastic responses when initial cues become unreliable, and thereby avoiding maladaptive responses (Grieco et al., 2002). Considering both past environmental conditions and the capacity of organisms for plasticity is thus essential for understanding how they might cope with changing environments.

Several studies have explored how past environmental conditions influence the responses of organisms to rising temperatures, often through a physiological framework. For example, the beneficial acclimation hypothesis (BAH) suggests that individuals acclimated to a given environment perform better in that environment than those acclimated to different temperatures (Leroi et al., 1994). Similarly, the optimal developmental temperature hypothesis (OTH) proposes that organisms acclimated to an optimal temperature maintain higher relative fitness across a range of temperatures than those acclimated to non-optimal conditions (Huey et al., 1999; Zamudio et al., 1995). Although these hypotheses have been investigated in various studies (e.g. Bennett and Lenski, 1997; Klepsatel et al., 2019; Steigenga and Fischer, 2009; Wilson and Franklin, 2002), the direct link between past thermal exposure and an individual's capacity for plasticity remains unexplored. Specifically, it is unclear whether individuals acclimated to distinct temperatures vary in their capacity for a plastic response when facing the same thermal challenge. This study addresses this gap by examining how past thermal conditions shape the extent to which individuals can adjust to temperature changes, building on – but not testing – the BAH and OTH frameworks.

For this purpose, we studied the thermal plasticity of the Acadian redfish, *Sebastes fasciatus*. At the adult stage, the species generally inhabits deep waters (250–500 m) of the temperate Northwest Atlantic Ocean (Gascon, 2003). This commercially valuable fish is experiencing rising ocean temperature. In the Estuary and Gulf of St Lawrence (hereafter St Lawrence System), the average maximum bottom temperature has increased from 5.2°C in 2009 to 6.9°C in 2023 (Galbraith et al., 2024). During the last decade, shifts in species distributions, changes in ecosystem productivity and an increase in *Sebastes* species biomass were observed both in the St Lawrence System (from 100,000 to 4,300,000 tonnes; Senay et al., 2023) and in other fisheries management areas of the Northwest Atlantic (DFO, 2023). The factors contributing to the increase in biomass of *Sebastes* spp. remain unclear, but rising temperature has been identified as a potential driving force, particularly affecting the early developmental stages within the St Lawrence System (Burns et al., 2020, 2021). This species has to deal with acute temperature challenges, both in its early development stages and as an adult. During development, while migrating to the seabed to continue its growth, juvenile *S. fasciatus* cross a cold intermediate layer at depths of ca. 50 to 120 m, characterized by temperatures around 0–1°C (Galbraith et al., 2024; Senay et al., 2023). As adult, *S. fasciatus* is found near the sea floor at temperatures ranging from 2.0°C to 7.0°C (Senay et al., 2023) and performs daily vertical migrations for feeding, during which they could be exposed to cooler temperature if approaching the cold intermediate layer at night (Gascon, 2003). Consequently, this species is likely to experience variable temperatures owing to these behaviors. The extent to which acclimation temperature influences the ability of this species to respond to acute temperature changes remains underexplored.

In this study, we not only assessed plasticity of the species following acclimation to different temperatures, but also investigated how thermal acclimation history influences the organism's capacity to respond to novel thermal changes. Plasticity applies for many phenotypic traits, from the molecular level to more integrated phenotypes encompassing physiological mechanisms, morphology and behavior. Here, we assessed the capacity for plasticity of the most fundamental level, i.e. gene expression, under warming ocean scenarios. Because we used a non-model species, we first characterized the molecular mechanisms underpinning the thermal plasticity of *S. fasciatus*. Then, we assessed the effect of two different acclimation temperatures on short-term (24 h) temperature exposure. Long-term (up to 10 months) temperature exposures mimic current (Galbraith et al., 2024) and predicted temperatures (Lavoie et al., 2020), while the short-term temperature changes simulate conditions that could be experienced by redfish during its daily vertical migration for feeding (Gascon, 2003). Functional analysis of differentially expressed transcripts also provided insight into the gene functions involved in the response of *S. fasciatus* to temperature variation.

## MATERIALS AND METHODS

### Experimental design

**Fig. 1.**
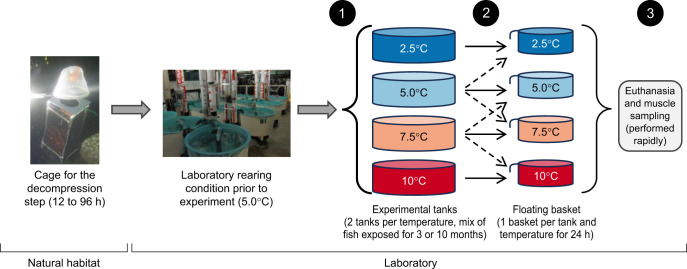
**Experimental design.** Flowchart illustrating the sequence of sample handling throughout the experiment. Fish were initially sampled from their natural habitat and placed in cages at a depth of 10–15 m to mitigate barotrauma. They were then transferred to the Maurice Lamontagne Institute, where they were maintained under the laboratory standard conditions at 5.0°C. The temperature experiment involved three key handling events for all treatments (indicated by number in black circles). (1) Fish were introduced into experimental tanks to begin long-term exposure, either at the start of the experiment (*T*_0_) for individuals exposed for 10 months, or at *T*_0_+7 months for those exposed for 3 months. (2) Twenty-four hours before the end of the experiment, a subsample of fish was transferred to floating baskets. Solid horizontal arrows indicate fish that remained at their original temperature (long-term exposure), whereas dashed oblique arrows represent fish transferred to a different temperature (short-term exposure). (3) At the end of the experiment, fish were euthanized and sampled for muscle tissue.

Tissue samples were collected from *Sebastes fasciatus* D. H. Storer 1854 in a tank experiment described in Guitard et al. (2025) and illustrated in Fig. 1. Briefly, fish were captured near les Escoumins, Québec, Canada (48.317801°N, 69.413287°W), in autumn 2019. SCUBA divers caught ca. 16–19 cm fish with dip nets at a depth of 25–30 m. This size range includes both immature and mature individuals (Brûlé et al., 2024). Fish were placed into cages (52×31×31 cm) that could hold up to 30 individuals, and full cages were left at a depth of 10–15 m depending on the tide for at least 12 h, but up to 96 h, to reduce barotrauma. Fish were then transported in oxygenated tanks to the Maurice Lamontagne Institute in Mont-Joli, Québec, Canada. At the time of capture, fish were likely residing below the seasonal thermocline, in a relatively cold and thermally stable environment. In early autumn, surface waters begin to cool, but deeper layers, where redfish typically occur, remain buffered from rapid temperature fluctuation owing to seasonal stratification and persistent presence of a cold intermediate layer (Galbraith et al., 2024). At the time of collection, temperature at these depths generally range between 2.0 and 7.0°C, with limited diel variability. Although less variable than sea surface waters, temperatures at 30–40 m still exhibit seasonal variation, typically ranging from near 0°C in winter to 6–7°C in late summer and early autumn, depending on local mixing dynamics. In addition, the region is influenced by tidally driven upwelling events, caused by strong currents and complex bottom topography, which can bring colder, deeper water to mid-depths (Bluteau et al., 2021; Cyr et al., 2015). These events may cause short-term temperature drops, even during warmer months, thereby shaping a thermal environment that is dynamic yet moderately variable.

We then conducted a long-term exposure experiment followed by a short-term exposure experiment. The experimental set-up was composed of eight circular tanks (1 m diameter; 760 liters) arranged into four temperature lines (2.5, 5.0, 7.5 and 10.0°C) and pH was maintained around 7.75 (total scale). The temperature range spans both below and above the species' typical habitat temperature (ca. 5°C), with the highest value (10°C) reflecting projections for the St Lawrence System by 2061 (Lavoie et al., 2020). On 9 and 10 December 2020, a total of 200 fish were anesthetized, weighed, measured and then randomly distributed across the eight experimental tanks (Fig. 1). Fish were acclimated to the experimental tanks for a week at the same rearing conditions (5°C). Then, temperatures were adjusted in increments of 0.5°C per day until all experimental temperatures were reached (6 January 2021). The end of the experiment was scheduled on 29 June 2021. However, slow growth rates and even losses of mass were observed during this 5 month experiment in all treatments, owing to a feeding protocol that did not result in *ad libitum* feeding and possibly a suboptimal fish density (Guitard et al., 2025). Thus, additional fish collected at the same time as the experimental fish, and kept in same rearing laboratory conditions prior to experiment, were introduced into each experimental tank on 13 July 2021. Note that no additional experimental procedures were performed on these fish, as they were solely included to serve as companions and enhance the well-being of the experimental fish. Since the experiment ended on 20 October 2021 (see below), this adjustment in the protocol resulted in long-term acclimation periods of either 287 or 99 days, corresponding to approximately 10 and 3 months, respectively, as referred to hereafter. The duration of the long-term experiment could be determined for each fish, as each individual was uniquely identified using a passive integrated tag.

To assess the short-term transcriptional response to temperature change, fish from 5.0 and 7.5°C treatments were subjected to a temperature shift of +2.5°C or −2.5°C for 24 h. All fish used in the long- or short-term exposure experiments, regardless of the subsequent temperature treatment, were treated the exact same way for the last 24 h of the experiment, ensuring standardized handling conditions across all experimental groups. The day before the end of the experiment, a circular floating basket (42 cm in diameter and 35 cm in depth) was installed in each experimental tank. The basket dimension allowed sufficient freedom of movement for minimal swimming. For fish acclimated to 5.0°C and 7.5°C, nine fish were randomly selected, briefly removed from the water, and quickly transferred according to the following: three fish were placed in the basket of the same tank, three fish were transferred to a basket in a 2.5°C lower temperature tank, and three fish were transferred to a basket in a 2.5°C higher temperature tank. To control for handling stress, three fish from the 2.5°C and 10.0°C treatments were randomly selected, briefly removed from the water, and placed in the basket within their original tank, mimicking the handling experienced by fish subjected to temperature changes. Baskets were used for all fish to minimize handling-induced stress, which has shown to be negligible after 24 h based on respirometry data (Guitard et al., 2025). Respirometry experiments using fish from the same experiment, but not selected for tissue collection, demonstrated that physical and handling stress only increased oxygen consumption for a few hours (Fig. S1) (Guitard et al., 2025). Furthermore, handling procedures were consistent across all treatments, ensuring that observed differences in transcriptomic responses could be attributed to temperature treatments rather than handling effects. Nevertheless, it remains possible that other physiological parameters could be influenced by the interaction between thermal exposure and handling stress, which we could not assess with our current experimental setup.

By the end of the experiment, all fish had reached a total length exceeding 20 cm and were sexually mature (Brûlé et al., 2024). On 20 October 2021, corresponding to 24 h after the final handling procedures, all fish from both the long- and short-term experiments were euthanized. Fish were removed individually from the floating basket and subjected to blunt force trauma, followed by exsanguination as part of the euthanasia procedure. This method was chosen to ensure rapid loss of consciousness and death, thereby minimizing physiological stress associated with prolonged euthanasia procedures, while also avoiding chemical agents that could interfere with blood chemistry and alter transcriptomic responses. A total of 48 fish were euthanized, which included 24 from the long-term exposure experiment (4 temperatures×2 tanks×3 fish) and 24 from the short-term exposure experiment (2 acclimation temperatures×2 acute temperatures×2 tanks×3 fish). For each individual, muscle tissue was sampled and flash-frozen in liquid nitrogen before being stored in −80°C until nucleic acid extraction. Genetic analysis confirmed that all individuals were *S. fasciatus* (Fig. S2A) and mostly belonged to a single genetic group (Fig. S2B; see Supplementary Materials and Methods for more details).

Fish collection in their natural habitat was performed under a Parks Canada permit (SAGMP-2019-33741). Sampling and experimental methods complied with the regulations of the Canadian Council on Animal Care and were approved by the Maurice Lamontagne Institute animal care committee (certificates 19-6B, 19-6C and 19-7B).

### RNA extraction, sequencing and bioinformatic pre-processing

RNA extraction of the 48 redfish was carried out on ca. 2 g of white muscle tissue, using Qiazol RNA extraction reagent (Qiagen), following the manufacturer's protocol. RNA-sequencing (RNA-seq) was used to investigate the changes in the entire transcriptome. Library construction using the NEB mRNA stranded library preparation kit and high-throughput sequencing steps were conducted by Génome Québec (Montreal, QC, Canada). Two sequencing batches were performed using Illumina NovaSeq 6000 S4 PE100 and PE150 with 33 and 21 libraries, respectively, including six samples sequenced in both batches. No sequencing batch effect on the measured transcript expression levels was detected (Fig. S3).

The RNA-seq raw reads quality was assessed using FastQC version 0.11.9 (https://www.bioinformatics.babraham.ac.uk/projects/fastqc/) and MultiQC version 1.9 (Ewels et al., 2016). They were then trimmed with Trim Galore! version 0.6.6 (https://www.bioinformatics.babraham.ac.uk/projects/trim_galore/) to remove adaptor sequences and to obtain a minimum Phred score of 30 and a minimum length of 20 bp. We aligned the trimmed reads to a *S. fasciatus* genome (BioProject accession number PRJNA1190455) using the splice-aware alignment program HISAT2 version 2.2.1 (Kim et al., 2015), with default parameters for paired-end reads.

*De novo* transcriptome reconstruction was performed with Stringtie version 2.2.1 (Pertea et al., 2015) based on the aligned reads for all libraries using a conservative mode. This procedure ensured complete identification of transcripts from the experimental samples. Structural annotation of the genome was performed using the Stringtie transcript merge usage mode to assemble transcripts from *de novo* transcriptomes from each library. This procedure resulted in the generation of a unified and non-redundant set of isoforms across the RNA-seq samples. We then quantified the number of reads per transcript with FeatureCounts version 2.0.3 (Liao et al., 2014) using the aligned reads from HISAT2 and the transcript annotation file generated by Stringtie transcript merge usage mode.

Gene Ontology (GO) assignments were used to classify the functions of *S. fasciatus* transcripts. The transcriptome functional annotation was obtained based on sequence homology to a known sequence database. NCBI's blastx tool version 2.12.0+ (Camacho et al., 2009) with an E-value cut-off of 0.001 was used to compare the transcript sequences with the SwissProt protein database (downloaded 30 May 2022). The UniProt information, including the associated GO terms associated with each hit, was then retrieved from the UniProt website.

### Statistical analysis

Gene expression variation partitioning and differential expression analyses were performed using the CRAN R package vegan version 2.6-4 (https://CRAN.R-project.org/package=vegan) and the Bioconductor package DESeq2 version 1.40.2 (Love et al., 2014). To quantify the proportion of the total gene expression variation explained by long- and short-term temperature exposures, we applied redundancy analysis (RDA; Borcard et al., 1992). First, samples were filtered based on exposure duration to differentiate the effect of long- and short-term temperature changes on gene expression. Long-term exposure conditions included individuals maintained at four temperatures for more than 3 months, whereas short-term exposure conditions encompassed individuals subjected to temperature shifts for 24 h. We used the normalized transcript count matrix (i.e. applying a variance stabilizing transformation to the number of reads per transcript matrix, as implemented in DESeq2) as the response variables. For long-term temperature exposure effects, the explanatory variable included the long-term temperature. Additionally, because fish could have been exposed for either 3 or 10 months to a given temperature (see experimental design described above), exposure duration was incorporated as a covariate in the RDA to assess its effect on gene expression (Table S1). For the short-term temperature change effects, we performed a distinct RDA using long- and short-term temperature exposures, and their interaction as explanatory variables (Table S1). In this case, fish were initially acclimated at 5°C or 7.5°C before been subjected to a temperature shift of +2.5°C or −2.5°C for 24 h.

We identified transcripts that showed changes in expression between temperatures by building a general linear model as implemented in DESeq2 and using the Wald test (Love et al., 2014). Transcripts with false discovery rate (FDR) <0.05 (*P*-value after Benjamini–Hochberg adjustment) and |log_2_FC|>1 were considered as differentially expressed. We used fish from the 5.0°C exposure as the reference temperature for the long-term exposure experiment, as they have been acclimated at this temperature from their arrival to the laboratory facilities until the experimental treatments. This procedure allowed us to test for differentially expressed transcripts (DETs) when temperature is lower (2.5°C) or higher (7.5°C or 10.0°C) than the reference temperature (5.0°C). Similarly, for the short-term temperature exposure treatment, redfish acclimated at 5.0°C or 7.5°C were used as reference conditions to test for DETs between long- and short-term temperatures, allowing us to identify transcripts involved in reduced (−2.5°C) or increased (+2.5°C) temperature within 24 h. DETs with similar expression patterns on the basis of Kendall rank correlations (at least 15 transcripts per cluster, correlation >0.7, testing the hypothesis of correlation for a confidence interval of 95%) were then grouped together, based on the normalized count matrix using the DEGreport version 1.36.0 package (http://lpantano.github.io/DEGreport/).

We used the GO assignments to classify the functions of *S. fasciatus* identified as DETs across temperatures. Enriched GO terms of the DETs were assessed using the ‘classic’ algorithm from the topGO version 2.52.0 package (https://bioconductor.org/packages/release/bioc/html/topGO.html) and based on *P*-values generated using Fisher's exact test method, with a threshold of FDR≤0.001 after Benjamini–Hochberg adjustment, for the three GO categories (i.e. biological process, molecular function and cellular component). To facilitate the interpretation of the obtained large list of GO terms associated with DETs, similar terms displaying the most probable gene function candidates were grouped based on their semantic similarity. The obtained reduced list of GO terms was then visualized using a treemap (space-filling visualization) of grouped terms using rrvgo version 1.12.0 (Sayols, 2023), where the GO terms function names were retrieved from the Bioconductor genome wide annotation for zebrafish *Danio rerio* (https://www.bioconductor.org/packages/release/data/annotation/html/org.Dr.eg.db.html). This procedure allowed us to visualize the relative significance of each function, where the area occupied by a given term is proportional to the −log_10_(FDR) (i.e. the larger is the space, the more statistically significant and probable is the gene function candidate). Finally, heat-shock proteins (HSPs), which are molecular chaperones that aid in protein folding and degradation, represent a widely conserved cellular stress response mechanism to temperature changes, and play an ecological and evolutionary important role in thermal adaptation (Feder and Hofmann, 1999; Logan et al., 2015; Tomanek, 2010). We specifically searched for transcripts annotated to HSP functions and assessed how temperature affected their expression in *S. fasciatus*.

## RESULTS

### *De novo* transcriptome reconstruction

We obtained a total of 2.26×10^9^ raw paired reads, from which 98.2% remained after the trimming step. The genome structural annotation was obtained based on the trimmed reads aligned on a *S. fasciatus* assembly, with an average overall alignment rate of 97.9% (s.d. 0.6%) per library. The *de novo* transcriptome reconstruction resulted in 35,483 putative transcript isoforms that could be grouped into 17,772 genes present across all samples, with a median length of 8619 bp. A total of 12,392 (69.7%) genes were characterized by a single transcript per gene. A total of 26,647 *S. fasciatus* transcripts (ca. 75.1% of 35,483 transcripts) were successfully annotated to a specific function based on the sequence homology to the SwissProt protein database and assigned to at least one GO term and categorized into a total of 14,285 GO terms.

### Transcriptional response to temperature variation

#### Quality check and sex effects on gene expression

FastQC quality check indicated that two libraries displayed a high proportion of overrepresented sequences (Fig. S4). A manual blast of these overrepresented sequences revealed that these two libraries displayed ribosomal RNA (rRNA) sequence contamination. This likely resulted from an incomplete or a failure of the rRNA depletion step during library preparation. These two libraries, which included one duplicated sample (i.e. sequenced twice, once in each of the two batches), were excluded from subsequent analyses. Additionally, from the remaining five duplicated samples, only one was randomly selected from each pair and retained. As a result, transcriptional responses to temperature variation were analyzed using a total of 47 libraries.

The sex of each individual was genetically assessed (Fig. S4A). No sex (adjusted *R*^2^=0.00%, *P*=0.669) or sex×temperature interaction (adjusted *R*^2^=0.00%, *P*=0.453) effects on transcript abundances were detected with the RDA (Fig. S5), indicating no sex-specific response to temperature change. As an additional validation step, including sex as a variable in the RDA did not alter the observed effect of temperature on gene expression (Table S1). Therefore, sex was not used as a covariate in the subsequent analyses.

#### Response to long-term temperature exposure

**Fig. 2.**
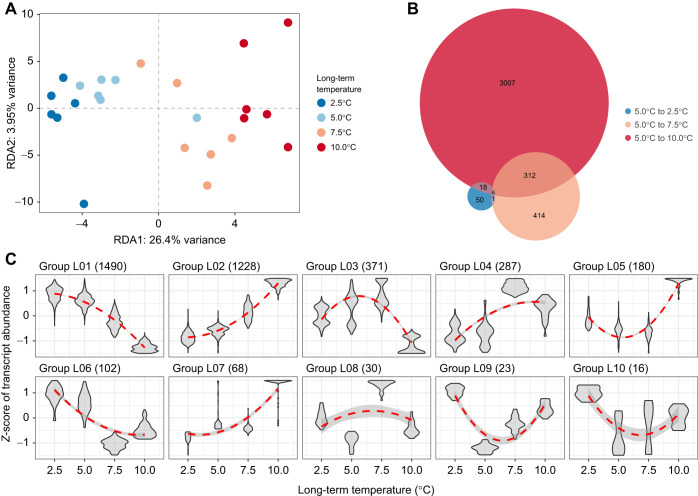
**Transcriptional response to long-term temperature exposure.** (A) Variation in transcript expression levels. Redundancy analysis (RDA) ordination plot showing the relationship between transcript expression levels and long-term temperature exposure, with colors indicating the different temperature treatments. (B) Number of differentially expressed transcripts (DETs). Venn diagram displays the count of DETs between the reference temperature (5.0°C) and the three other long-term temperature exposures, 2.5°C (blue), 7.5°C (orange) and 10.0°C (red). (C) Groups of co-expressed transcripts for DETs among long-term temperature exposures. Numbers in parentheses indicate the number of transcripts belonging to each co-expression module.

Transcript levels differed among the long-term temperature exposure treatments (adjusted *R*^2^=22.67%, *P*=0.001; Fig. 2A). Fish exposed to 2.5°C and 5.0°C exhibited similar transcriptomic profiles, whereas those exposed to 10.0°C showed the most distinct transcriptomic profiles compared with the lower temperature treatments. Fish maintained at 7.5°C exhibited transcriptomic profiles that were intermediate between those of fish exposed to 2.5°C, 5.0°C and 10°C (Fig. 2A). Pairwise comparison of DETs supported this pattern: the fewest DETs were observed between 2.5°C and 5.0°C treatments (*n*=75 DETs; Fig. 2B), whereas substantially more DETs were identified between 5.0°C and 7.5°C (*n*=733 DETs; Fig. 2B), and between 5.0°C and 10.0°C (*n*=3343 DETs; Fig. 2B). Notably, no significant differences in transcript levels were detected between fish exposed for 3 versus 10 months (adjusted *R*^2^=1.05%, *P*=0.102), and both conditions were thus considered as long-term acclimation.

DETs among temperatures for long-term exposure were grouped into 10 groups of co-expressed transcripts (see Functional analysis of DETs, below). More than 71% of these DETs displayed an almost linear effect, either decreasing (group L01, *n*=1490 DETs, 39.26%; Fig. 2C) or increasing (group L02, *n*=1228 DETs, 32.36%; Fig. 2C) transcript expression levels as a function of temperature. For these two groups of DETs, transcript expression levels were again more similar for fish exposed to 2.5°C and 5.0°C compared with fish at higher temperature exposures. Another similar expression pattern was observed for the DET groups L04 (*n*=287, 7.56%) and L06 (*n*=102, 2.69%), where a step increase and decrease in transcript expression level was detected at 7.5°C, respectively. This result showed that decreasing temperature from 5.0°C to 2.5°C triggers fewer gene expression changes than increasing temperature from 5.0°C to 7.5°C. The expression pattern observed in DETs between temperatures from groups L03 (*n*=371), L05 (*n*=180), L09 (*n*=23) and L10 (*n*=16), representing 15.55% of DETs among long-term temperature exposures, was characterized by either a U- or bell-shaped expression pattern (Fig. 2C) where higher or lower expression levels, respectively, were observed for fish from extreme temperatures (i.e. 2.5°C and 10.0°C) compared with those from intermediate ones (i.e. 5.0°C and 7.5°C).

#### Response to short-term temperature exposure

**Fig. 3.**
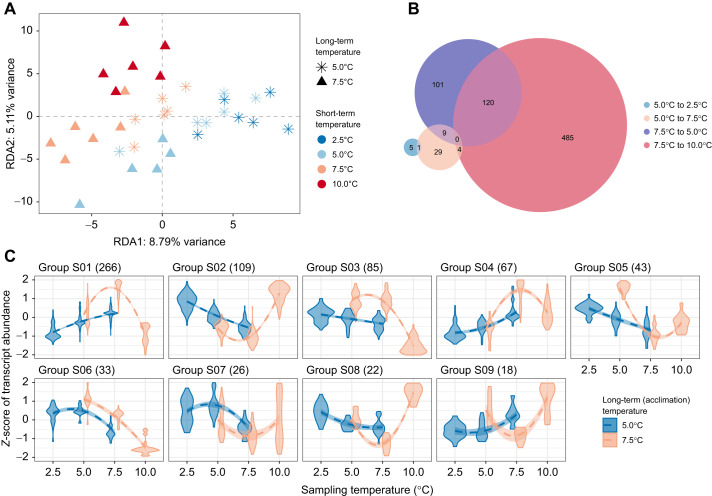
**Transcriptional response to short-term temperature exposure.** (A) Variation in transcript expression levels. RDA ordination plot showing the relationship between transcript expression levels and temperature treatments, with point shape indicating long-term temperature exposures and colors representing short-term temperature exposures. Different long- and short-term temperatures indicates that individuals were subjected to a short-term temperature change. (B) Number of DETs. Venn diagram displays the count of DETs between the acclimation temperature (5.0°C or 7.5°C) and sampling temperature [2.5°C (blue) and 7.5°C (orange) for fish acclimated at 5.0°C, and 5.0°C (purple) or 10.0°C (red) for fish acclimated at 7.5°C]. (C) Groups of co-expressed transcripts for the total DETs obtained in B, represented according to sampling temperatures and grouped by acclimation conditions (colors). Numbers in parentheses indicate the number of transcripts belonging to each co-expression module.

Transcript expression levels were also affected by the short-term temperature exposure. Variation partitioning analysis revealed that both the long-term exposure (i.e. where fish spent up to 10 months; adjusted *R*^2^=3.43%, *P*=0.005) and the short-term exposure (where fish spent 24 h after the temperature change, adjusted *R*^2^=2.49%, *P*=0.008) significantly contributed to variation in transcript abundance (Fig. 3). The response to temperature changes was asymmetrical as a function of the direction of temperature changes. For fish from long-term exposure conditions at 5.0°C and 7.5°C, a greater number of DETs were detected in fish subjected to a temperature increase of 2.5°C compared with those exposed to a temperature decrease of 2.5°C (Fig. 3B). Interestingly, we observed that changes in expression levels of certain genes occurring withing the first 24 h were sustained over a period of 10 months. This pattern was particularly evident in groups S01, S04 and S08 at 5.0°C, and S02, S05 and S07 at 7.5°C (Fig. 3C).

The long-term×short-term temperature interaction also explained a statistically significant, though modest, proportion of the variation in transcript abundance (adjusted *R*^2^=2.49%, *P*=0.020). The influence of long-term acclimation in the short-term response to temperature shift is evidenced by the significantly fewer DETs among sampling temperatures for fish acclimated at 5.0°C (*n*=48 DETs) compared with those acclimated at 7.5°C (*n*=719 DETs; Fig. 3B). This was also observed at the level of transcript reaction norms (Fig. 3C). For DETs associated with the short-term specific response, fish acclimated at 5.0°C showed relatively linear reaction norms (Fig. 3C, blue lines). In contrast, the majority of reaction norms for fish acclimated at 7.5°C were characterized by bell- or U-shaped patterns. This indicates a similar gene expression response to either a 2.5°C increase or decrease in temperature (all except S06; Fig. 3C).

### Functional analysis of DETs

We used a GO enrichment analysis to examine whether the transcriptional plasticity in functional response differed between long- and short-term temperature changes in *S. fasciatus*. We investigated the function of different groups of DETs: those exclusive to long-term response (*n*=3501 DETs), those common to both long- and short-term response to temperature changes (*n*=307), and those exclusive to short-term response. For the short-term response, we further differentiated between DETs detected in samples acclimated at 5.0°C (*n*=31) and 7.5°C (*n*=428; Fig. S6), which allowed us to better assess the gene function associated with the significant long-term×short-term temperature interaction.

**Fig. 4.**
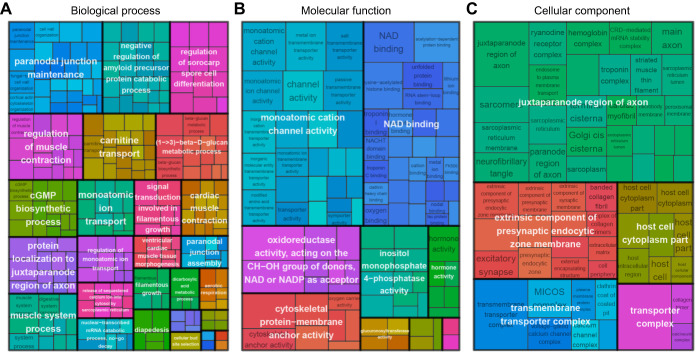
**Gene Ontology (GO) terms associated with exclusively long-term response to temperature change.** Treemap for (A) biological process, (B) molecular function and (C) cellular component, where GO terms were grouped (color) based on their semantic similarity, and the space used by the term is proportional to the −log_10_(adjusted *P*-value), hence the gene function candidate probability.

DETs associated with long-term response to temperature changes primarily involved in the regulation of muscle activity including the regulation of muscle contraction, muscle tissue morphogenesis and cell physiological processes like ion transport, cGMP biosynthesis or amino-acid transport (Fig. 4). We also performed a GO enrichment analysis on groups of co-expressed that displayed a downregulation (group L01; Fig. 2C) or upregulation (group L02; Fig. 2C) under higher temperature conditions. Genes associated with transmembrane transport, muscle contraction, oxidoreductase and hormone activities were downregulated (Fig. S7), whereas genes involved in cell growth and organization, protein folding or biogenesis were upregulated (Fig. S8) at warmer temperatures. Interestingly, we also observed higher expression of transcripts associated with histone H3R17 methyltransferase activity (Fig. S8B) at higher temperatures, suggesting fine-tuning of gene expression regulation via epigenetic processes.

**Fig. 5.**
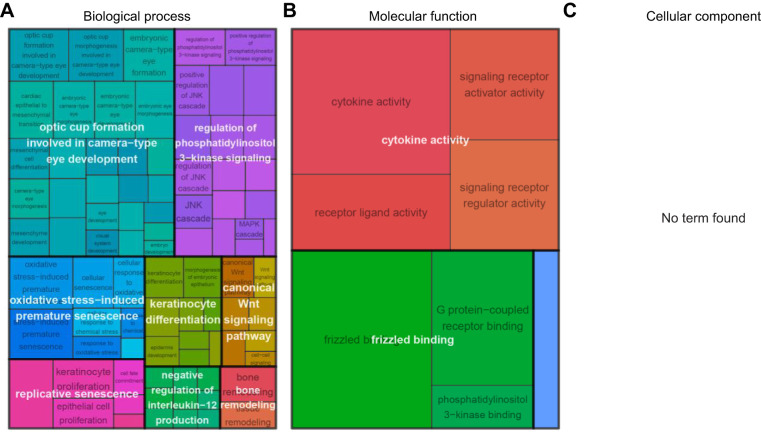
**GO terms associated with DETs in common between long- and short-term response to temperature change.** Treemap for (A) biological process, (B) molecular function and (C) cellular component, where GO terms were grouped (color) based on their semantic similarity, and the space used by the term is proportional to the −log_10_ (adjusted *P*-value), hence the gene function candidate probability.

DETs shared between long- and short-term temperature responses involved genes associated with stress responses. This included mainly gene functions linked to stress-induced cellular senescence and cell communication (Fig. 5). Although the tissue used in the present study was white muscle, we still observed a GO enrichment for the optic cup formation for eyes development (Fig. 5A). This represented a group of 143 DETs, distributed among 26 reduced GO terms. These DETs were involved mostly in the long- and short-term temperature response for samples acclimated at 7.5°C, representing 116 and 11 DETs, respectively. They also contributed to a common response between the long- and short-term temperature response for fish acclimated at 7.5°C, with 15 common DETs between both conditions, whereas the short-term temperature response for fish acclimated at 5.0°C only involved one DET associated with this eye development biological process.

**Fig. 6.**
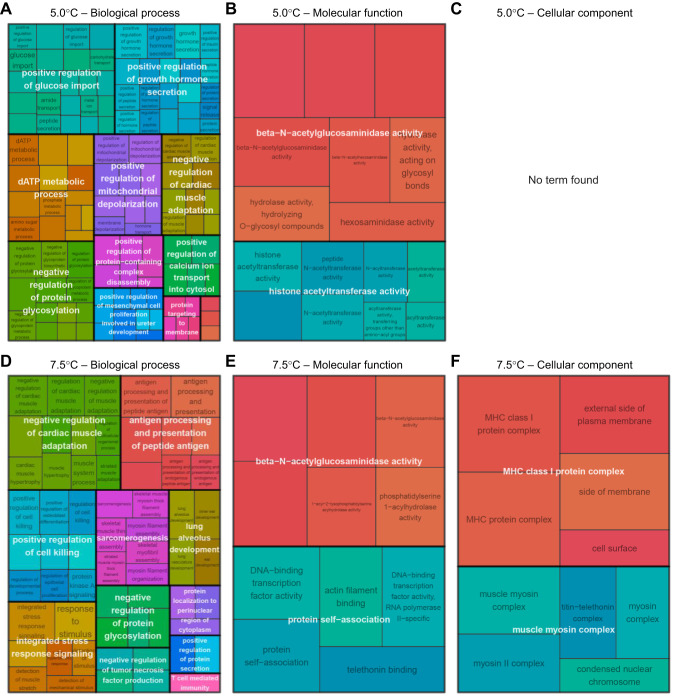
**GO terms associated with exclusively short-term response to temperature changes, for fish acclimated at different temperatures.** (A–C) 5.0°C; (D–F) 7.5°C. Treemap for (A,D) biological process, (B,E) molecular function and (C,D) cellular component, where GO terms were grouped (color) based on their semantic similarity, and the space used by the term is proportional to the −log_10_(adjusted *P*-value), hence the gene function candidate probability.

Gene functions exclusively associated with short-term temperature change were found to depend on the long-term acclimation temperature. Samples acclimated at 5.0°C involved DETs associated with energy production (e.g. glucose import, hormone secretion, dATP metabolic process; Fig. 6A), and gene expression regulation (e.g. histone acetyltransferase; Gong et al., 2020; Fig. 6B). Samples acclimated at 7.5°C also expressed genes linked to muscular cell and gene regulation but they also mainly displayed stress-related gene expression, with DETs associated with cell signaling, immune cell communication and cell killing regulation (Fig. 6D–F). Specifically, the search of the key word ‘stress’ in GO term enrichment results revealed a total of eight GO terms associated with response to temperature fluctuation for fish acclimated to 7.5°C, whereas no GO terms with the keyword ‘stress’ were detected for those acclimated to 5°C.

A targeted search for the activity of HSPs revealed that this gene family is indeed present and expressed in *S. fasciatus* but exhibits a weak response to temperature changes. We identified a total of 125 transcripts associated with HSPs. Of these, only 25 were found to be differentially expressed in response to long-term temperature exposure, whereas the majority (100 transcripts) maintained uniform expression levels across all four experimental temperatures (Fig. S9).

## DISCUSSION

In this study, we described the transcriptional plasticity in redfish *S. fasciatus*, in response to both long- and short-term temperature changes. Gene expression responded to both durations of temperature exposure, highlighting the thermal plasticity of *S. fasciatus*. Plasticity in gene expression allowed the species to cope with temperatures exceeding those typically encountered in their habitat within the St Lawrence System and close to the maximum temperature at which the species has been observed in the wild (13°C in the Gulf of Maine; Scott, 1982). Gene expression was maintained and changed rapidly for long- and short-term exposures, respectively, highlighting the capacity of this species for a plastic response to temperature changes. Multiple gene functions were implicated in both long- and short-term responses, including metabolic, protein synthesis and stress-related pathways. Prior thermal experience also influenced the response to acute temperature change. Fish acclimated to warmer temperatures displayed more short-term transcriptomic changes that were associated with more stress-related gene functions compared with those acclimated at a colder temperature. Our analysis of gene expression responses to temperature stress reveals the molecular mechanisms underlying thermal plasticity in *S. fasciatus*, offering insight into its potential sensitivity to future ocean warming. Despite evidence of transcriptional plasticity, our findings suggest that *S. fasciatus* may still experience physiological stress under projected ocean warming scenarios in the warmest part of its distribution, such as the Gulf of St Lawrence. This suggests that plasticity alone may not be sufficient to shield the species from the impacts of climate change.

### The plasticity of gene expression responses to long- and short-term temperature exposures

Reaction norms, which characterize the range of trait changes across environmental conditions, describe an organism's capacity for plasticity. In this study, transcript expression patterns were distinct among fish exposed to four temperatures, illustrating the capacity for transcriptional thermal plasticity in *S. fasciatus*. The near-linear expression profiles relative to the temperature gradient suggest a progressive physiological adjustment to warming. Although plasticity is widely recognized among fish species in response to environmental change (reviewed in Oomen and Hutchings, 2017), our study provides a detailed characterization of the gene expression changes associated with long-term thermal acclimation. Specifically, we observed shifts in expression across pathways related to metabolism, protein synthesis and stress responses, patterns consistent with those reported in other species (Logan and Somero, 2010; Manzon et al., 2022; Metzger and Schulte, 2018; Windisch et al., 2014).

The long-term temperature exposure experiment revealed that transcript profiles were more similar among fish acclimated to 2.5°C and 5.0°C, whereas greater differences were observed between fish acclimated to 5.0°C and higher temperatures (7.5°C and 10°C). Interestingly, these transcriptional responses parallel whole-fish performance and align with a recent study demonstrating the optimal growth of this species at 2.5°C and 5.0°C, temperatures that sustain a manageable metabolic demand (Guitard et al., 2025). This temperature range also reflects the temperature typically encountered by this species in its natural habitat (Gascon, 2003; Senay et al., 2023). Altogether, these results suggest that 2.5°C to 5.0°C could represent the species' preferred temperature range.

Assessing the speed at which organisms adjust their physiological, behavioral or molecular traits in response to environmental changes is crucial to our understanding of the adaptive potential of plasticity in the face of shifting conditions (Burton et al., 2022; Dupont et al., 2024; Einum and Burton, 2023). Our results from the short-term exposure revealed that *S. fasciatus* responded to temperature variations within a 24-h timeframe, highlighting the ability of this species to rapidly modulate gene expression. These findings are consistent with the rapid response to acute thermal changes observed in various fish species (Logan and Somero, 2010; Long et al., 2012; Shi et al., 2019; Thorne et al., 2010; Tomalty et al., 2015). We also observed that gene expression levels associated with specific thermal conditions were comparable between short-term (24 h) and long-term (up to 10 months) exposures to 5.0°C and 7.5°C. Notably, the linear reaction norm established within 24 h for fish acclimated to 5.0°C persisted after 10 months, and no significant differences were found in gene expression between individuals exposed for 3 versus 10 months. These results suggest that the transcriptional response to temperature change was rapidly initiated and remained stable over time, as long as temperature remained constant. This has important implications for understanding how fish may respond to seasonal or prolonged thermal events, such as multi-month marine heatwaves (Hutchings et al., 2007; Oomen and Hutchings, 2017; Vagner et al., 2019). We did not assess whether these expression patterns are adaptive, but the consistency across time points indicates that *S. fasciatus* has the capacity to mount and sustain transcriptional responses to thermal variation. This may reflect the underlying mechanisms of plasticity relevant to long-term environmental exposure. Moreover, the ability to rapidly initiate gene expression responses to temperature shift could be adaptive in a fluctuating environment. Under global change scenarios, such rapid plasticity may reduce the duration an organism experiences mismatches between phenotype and environment, potentially mitigating fitness cost with suboptimal conditions (Dupont et al., 2024; Padilla and Adolph, 1996).

### Acclimation environments drive the response to acute temperature changes

Besides the temporal dynamics of phenotypic adjustment, it is also essential to consider the temporal variations of environmental conditions. Although the predictability of environmental changes has been largely explored when studying plasticity evolution (Botero et al., 2015; Gavrilets and Scheiner, 1993; Leung et al., 2020; Reed et al., 2010; Tufto, 2015), and the effect of temperature on transcriptional response has been examined by numerous studies (Buckley et al., 2006; Gracey et al., 2004; Healy et al., 2017; Jayasundara et al., 2013; Logan et al., 2015; Logan and Somero, 2010; Oomen and Hutchings, 2017; Podrabsky and Somero, 2004; Wellband and Heath, 2017), the role of acclimation environment in shaping the capacity for plasticity has received comparatively less attention (but see Bay and Palumbi, 2015; Logan and Somero, 2011). In the present study, we not only assessed transcriptional plasticity following acclimation history, but also investigated how acclimation history influences an organism's ability to respond to new temperature changes. After exposing all fish for more than 3 months to one of four temperatures, some fish were then subjected to an acute temperature shift to evaluate the resulting capacity for plasticity. This allowed us to assess how prior acclimation affects their ability to respond dynamically to the same temperature variations.

Thermal acclimation can influence an organism's response to acute temperature changes, including alteration in mitochondrial respiration rates and shifts in the shape of the thermal performance curve (Baris et al., 2016; Schulte et al., 2011). This phenomenon has also been investigated in fish at the level of gene expression plasticity, highlighting the molecular mechanisms underlying thermal adaptation. For instance, Logan and Somero (2011) demonstrated that in the goby *Gillichthys mirabilis*, thermal acclimation shifted the threshold for initiating the stress-related gene expression response during acute heat stress. Specifically, warm-acclimated individuals delayed their transcriptional response until more extreme temperatures were reached (Logan and Somero, 2011). This suggests that acclimation modulated the timing of the stress response. In contrast, our results indicate that in *S. fasciatus*, thermal acclimation did not delay the onset of transcriptional responses. Instead, individuals exhibited distinct gene expression profiles depending on their acclimation temperature, suggesting that acclimation influenced the nature or composition of the response rather than a shifting threshold.

Short-term temperature exposures suggested that the population performed better when acclimated to 5.0°C compared with 7.5°C, based on both the shape of the reaction norms and the functional profiles of the DETs. Specifically, fish acclimated to 5.0°C exhibited a linear reaction norm, whereas those acclimated to 7.5°C showed a bell- or U-shaped reaction norm, suggesting a more limited capacity for plasticity at the higher acclimation temperature. This difference may also reflect variation in thermal performance, with the bell shape response at 7.5°C acclimation temperature resembling classic thermal performance curves in physiological studies. Such curves are typically characterized by a slow initial increase in performance with rising temperature, a peak at an intermediate temperature, followed by a rapid decline at higher temperature (Angilletta, 2009; Huey and Stevenson, 1979). In our study, this pattern in the reaction norm of fish acclimated to 7.5°C suggests a reduced capacity of *S. fasciatus* to tolerate deviations of +2.5°C or −2.5°C from this acclimation temperature, compared with individuals acclimated to 5.0°C. This interpretation is further supported by the higher number of stress-related transcripts in warm-acclimated fish during short-term temperature challenges, which may reflect a higher physiological cost. In contrast, cold-acclimated fish showed gene expression changes more closely associated with growth-related processes, potentially reflecting a more favorable physiological state. This interpretation is also consistent with previous findings showing optimal growth performance in *S. fasciatus* at long-term acclimation temperatures of 2.5°C and 5.0°C, compared with 7.5°C and 10.0°C (Guitard et al., 2025).

Our results suggest that acclimation alone may not be sufficient to fully prepare *S. fasciatus* to cope with projected global warming scenarios. Over the past 15 years, the temperature experienced by *S. fasciatus* in the St Lawrence System has increased from ca. 5–6°C to ca. 7.0°C (Lavoie et al., 2020). In the future, *S. fasciatus* populations could experience the range of temperature variation tested in this experiment in its natural habitats due to the ongoing warming of the St Lawrence System (Galbraith et al., 2024). Additionally, short-term temperature changes (+2.5°C or −2.5°C) occur as a result of their developmental or daily feeding vertical migrations (Gascon, 2003). Our findings suggest that further warming, as expected with climate change, may surpass the capacity of this species to respond through plasticity alone. Although *S. fasciatus* exhibits transcriptional plasticity, the elevated expression of stress-related genes in warm-acclimated individuals and the bell- and U-shaped reaction norms observed for fish acclimated to 7.5°C suggest potential physiological limits. This implies that rising ocean temperatures could not only impact the phenotype expressed during acclimation, but also limit an organism's plastic potential, thereby affecting its capacity to cope with environmental variability. Consequently, other mechanisms, such as behavioral thermoregulation, habitat shifts or migration, may become increasingly important for ectothermic organisms facing extreme or prolonged thermal stress (Gunderson and Stillman, 2015).

### Genetic variation in phenotypic plasticity

Within the St Lawrence System, *S. fasciatus* exhibits notable genetic diversity. A recent population genomic study has identified at least three distinct populations, with instances of multiple populations being captured in a single bottom-trawl set (Benestan et al., 2021). In our study, we conducted these experiments with animals captured in the St Lawrence Estuary that were not studied in Benestan et al. (2021). Thus, we used ddRAD-seq genotypes from the *S. fasciatus* studied for transcriptomics and other *S. fasciatus* from the St Lawrence System to understand whether the individuals used in this study were associated with one or multiple populations (Supplementary Materials and Methods). The specimens for this transcriptomic study formed a relatively genetically homogeneous group, distinct from other genetic groups of *S. fasciatus* observed in the St Lawrence System (Fig. S2). The adaptive genetic variance may differ among these *S. fasciatus* genetic groups or populations. It is plausible that some populations have different thermal tolerance than the *S. fasciatus* population we tested. Therefore, the potential for local adaptation to cope with increasing temperature is a valid consideration. For example, a previous study of population genetics identified a distinct population in the Gulf of Maine (Valentin et al., 2014), where individuals encounter areas with temperatures reaching up to 13.0°C (Scott, 1982).

The capacity for phenotypic plasticity also has a genetic basis, leading to variation in plastic responses among genetically diverse individuals and providing a source for natural selection to act upon, underscoring its evolutionary significance (Gavrilets and Scheiner, 1993; Landy et al., 2020; Leung et al., 2023; Scheiner, 1993). Recent evidence shows that the temporal dynamics of plasticity, the time required for an organism to adjust its phenotype in response to environmental changes, have diverged among different classes of ectotherms (Einum and Burton, 2023). This demonstrates that both the capacity for plasticity and the speed at which plastic responses are expressed are evolving traits, with important implications for evolutionary ecology. Therefore, unraveling the genetic underpinnings of phenotypic plasticity, including both its capacity and temporal dynamics, will provide valuable insights into how species adapt to changing environment, and the potential strategies they may employ for survival. The genetic variation observed in *S. fasciatus* natural population may thus reflect differences in both the extent and timing of plastic responses.

### Gene functions involved in short- and long-term responses

Mechanisms underlying responses to temperature changes in stenothermal fish are not well understood (Ern et al., 2023). Our analysis of gene functions sheds light on the molecular response to fluctuating temperature for *S. fasciatus*, a temperate and stenothermic species. Long- and short-term temperature exposures identified epigenetic, stress-related and heat coping molecular mechanisms associated with thermal responses. Our study also identified a small proportion of HSPs along with a number of genes associated with non-muscle-related functions, such as eye development, lung alveolar formation and bone remodeling, that contribute to the muscle transcriptomic response to temperature changes in *S. fasciatus*.

For both long- and short-term responses to temperature change, GO analysis revealed rapid involvement of epigenetic processes, in particular those implicated in chromatin rearrangement via histone acetylation (Turner, 2000) and protein folding (Azzaz and Fantini, 2022; Harvey et al., 2018). Epigenetic mechanisms are defined as any chemical modifications that regulate gene expression pattern without any change in DNA sequence. The epigenome (all epigenetic modification of the genome of an organism) could be environmentally responsive and has thus been proposed as a major mechanism underlying phenotypic plasticity (Angers et al., 2010; Bollati and Baccarelli, 2010; Leung et al., 2023; Simola et al., 2016). The extent to which an organism can respond to current and future temperature extremes via epigenetic processes could influence its capacity to cope with global change (Bogan et al., 2020; Rey et al., 2020).

The stress-related functions involved in both long- and short-term responses to temperature variation highlight the physiological demands imposed on this species by rising temperatures. This is clearly demonstrated by the higher prevalence of stress-related functions in the short-term response, particularly in fish acclimated at the highest temperature. After 24 h of exposure, response to temperature changes were mainly characterized by stress-related and energy production functions. Short-term temperature changes typically involve HSPs in fish (Jeffries et al., 2016; Liu et al., 2013; Tomanek, 2010). In the present study, out of 125 transcripts identified as belonging to the HSP gene family, only a few (*N*=25) exhibited a heat shock response, whereas the majority were expressed at the same level across all experimental temperatures. The thermal niche of *S. fasciatus* is currently unavailable. Although it could be inferred from population distributions in the field, our results suggest it is narrow, at least for this population from the St Lawrence System. This interpretation is supported by the stable expression of HSP genes, which may reflect a constitutive protective mechanism rather than a response to acute thermal stress. A similar pattern has been observed in Antarctic species, where constant HSP expression helps mitigate the elevated levels of protein damage in cold environments (Clark et al., 2008a; Place et al., 2004; Place and Hofmann, 2005). Moreover, variation in expression patterns and levels of induction could also be observed among *HSP70* gene isoforms, as described in the clam *Laternulla elliptica* (Clark et al., 2008b). Our results also suggest the presence of other heat coping molecular mechanisms, akin to the function of HSPs. We observed that genes associated with cell growth and organization, protein folding or biogenesis were upregulated at higher temperatures, whereas cell communication, transmembrane transport and oxidoreductase activity genes were downregulated. These functions suggest a role similar to that of HSPs, which are known to assist in refolding damaged proteins (Feder and Hofmann, 1999).

We identified non-muscle-related gene functions that responded to both long- and short-term temperature changes in *S. fasciatus* muscle tissues. This may reflect gene duplication and diversification, or a pleiotropic role of the same gene. For example, rhodopsin, typically annotated as an eye-specific gene, is a highly conserved, heat-sensitive gene also expressed in vertebrate musculoskeletal tissues (Kojima and Sudo, 2023). It has been linked to depth adaptation in marine fishes, with evidence of positive selection across multiple species, including *Sebastes* spp. (Larmuseau et al., 2011; Pampoulie et al., 2015; Schott et al., 2014; Sivasundar and Palumbi, 2010). Although often classified as ‘vision-associated’, many of these genes perform diverse functions across tissues and developmental stages. For instance, in zebrafish, opsin gene diversity, likely resulting from duplications, has been associated with non-visual roles, such as circadian regulation, light-seeking behaviors and seasonal responses (Davies et al., 2015). However, the functions of many non-visual opsin genes remain poorly understood. Although most current research focuses on their expression in eye tissues (e.g. Boyette et al., 2024), our findings suggest that these genes may also contribute to thermal sensing or stress responses in muscle. Similarly, the *six6* gene, initially characterized for its role in eye development, has been shown to have pleiotropic functions across tissues and developmental stages, including sensory and ecological adaptation in Atlantic salmon, *Salmo salar* (Moustakas-Verho et al., 2020).

### Conclusions

Our study provides valuable insights into the molecular basis of thermal plasticity and how *S. fasciatus* responds to environmental variability, emphasizing the need to consider the temporal dynamics of both environmental changes and phenotypic adjustment. We show that fish acclimated to higher temperatures exhibit increased stress-related gene expression in response to subsequent temperature shifts, suggesting that prior environmental exposure can shape the capacity for plasticity and the nature of future responses. This highlights the role of thermal history in modulating plastic responses and suggests that plasticity itself may be constrained under chronic environmental stress. Moreover, the direction of temperature change appears to be critical: cold exposure resulted in milder transcriptional responses than warm exposure, suggesting that temperature decrease may help mitigate stress, lower the risk of hypoxia by reducing oxygen consumption, and improve survival outcomes. These findings underscore the practical relevance of thermal plasticity research for fisheries management and conservation. Finally, by integrating transcriptomic data across different timescales, our work enhances the predictive value of plasticity research for assessing species resilience and vulnerability in a warming ocean by providing new insights into the molecular mechanisms involved in thermal plasticity.

## Supplementary Material

10.1242/jexbio.251288_sup1 Supplementary information
