# Supplementary material for: Past environments modulate response to fluctuating temperatures in a marine fish species, Sebastes fasciatus
Source: J Exp Biol. 2025 Nov 4;228(21):jeb251288. doi: 10.1242/jeb.251288 (PMC12633736; doi:10.1242/jeb.251288)
Supplement: Supplementary information [file jexbio-228-251288-s1.pdf]

Supplementary Materials and Methods

Population genetic structure analysis

The DNA of 72 *Sebastes* from the basin experimental design (including the 48 used in the transcriptomic experiment) was extracted from muscle with DNeasy Blood and Tissus or DNeasy 96 (Qiagen) and quantified on a Synergy LX (BioTek) using PicoGreen to confirm high quality DNA extracts. Double digest restriction-site-associated DNA (ddRAD; *PstI* and *MspI* enzymes) libraries were prepared by the Plateforme d'Analyse Génomique (IBIS, Université Laval) using 20 ng of DNA per sample following Poland et al. (2012). Libraries were sequenced on a NovaSeq 6000 S4 150 PE at Genome Quebec, with 10% PhiX.

Genotyping was performed using STACKS modules (v.2.55, (Catchen et al. 2013; Rochette et al. 2019). Read quality was checked using FastQC (Andrews 2010) and multiQC (Ewels et al. 2016), and Illumina adaptors were removed with Trimmomatic (Bolger et al. 2014). Reads were demultiplexed with *process_radtag* module, with a truncation at 135 pb, then aligned to a *Sebastes fasciatus* genome (Genbank accession: JBJQUQ000000000) using BWA-MEM (Li and Durbin 2009) with default parameters.

We used the *gstacks* module to create two different SNP catalogs, 1) the species catalog to confirm that all individuals were *S. fasciatus* and 2) the population catalog to check for individual difference linked to population structure. To reach these objectives, we included in our analysis individuals from a fine scale population structure ddRAD project with similar DNA extraction and library preparation methods (Bourret et al. in prep). For the species catalog, we added 97 individuals representative of *S. fasciatus*, *S. mentella* and *S. norvegicus*. For the population catalog, we added 67 individuals presentative of the genetic diversity within *S. fasciatus*. From these two catalogs, different filtration steps were performed to keep only SNPs shared by > 75% individuals (*population* modules, r = 0.75, MAF 0.05), low missingness SNPs (<10%), to remove SNPs with observed heterozygosity (H_O) > 0.6 and to keep only 1 SNP by loci. The resulting species and population dataset comprised 35,910 SNPs and 10,863 SNPs, respectively. Population structure was assessed using principal component analysis (PCA) using the *glPCA* function of adegenet R package (Jombart et al. 2008). All the individuals from the tank experiment, including the 48 transcriptomic ones, were confirmed to be *S. fasciatus* (Fig. S2A), and most of them were genetically similar (Fig. S2B).

Journal of Experimental Biology • Supplementary information

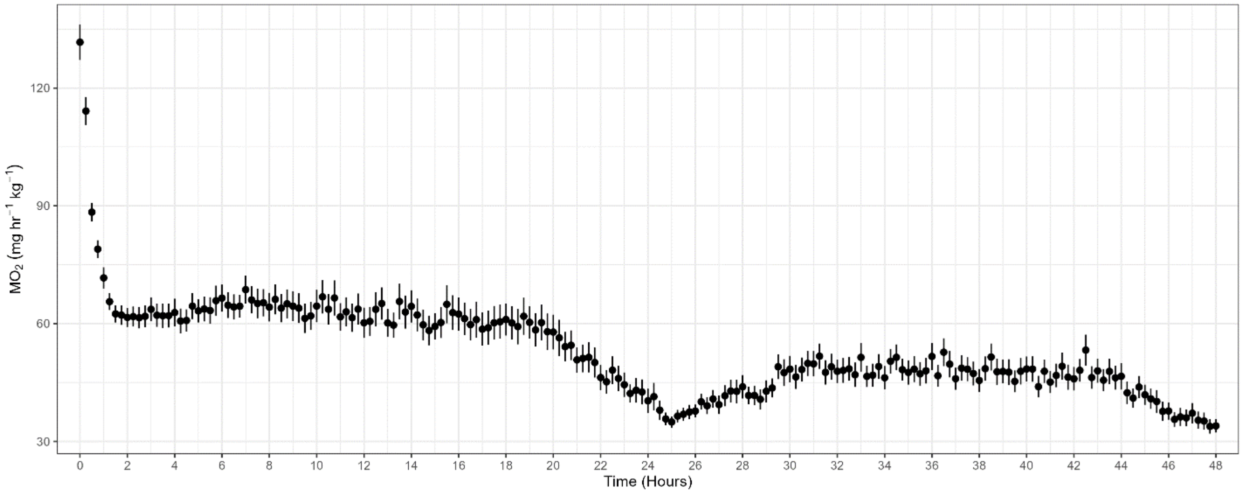

Fig. S1. Time course of oxygen consumption after handling. After a physical stress and handling involved in an experiment to assess both standard and maximum metabolic rates, oxygen consumption (MO_2) was monitored over a 48-hours period. Oxygen consumption was initially elevated due to handling stress but declined rapidly within the first hour. From the second hour onward, the decline in MO_2 continued gradually, reaching near-standard MO_2 levels by 24 h post-handling. Each point indicates the mean MO_2 of 62 individuals, with vertical bars indicating standard error. Data adapted from Guitard et al. (2025).

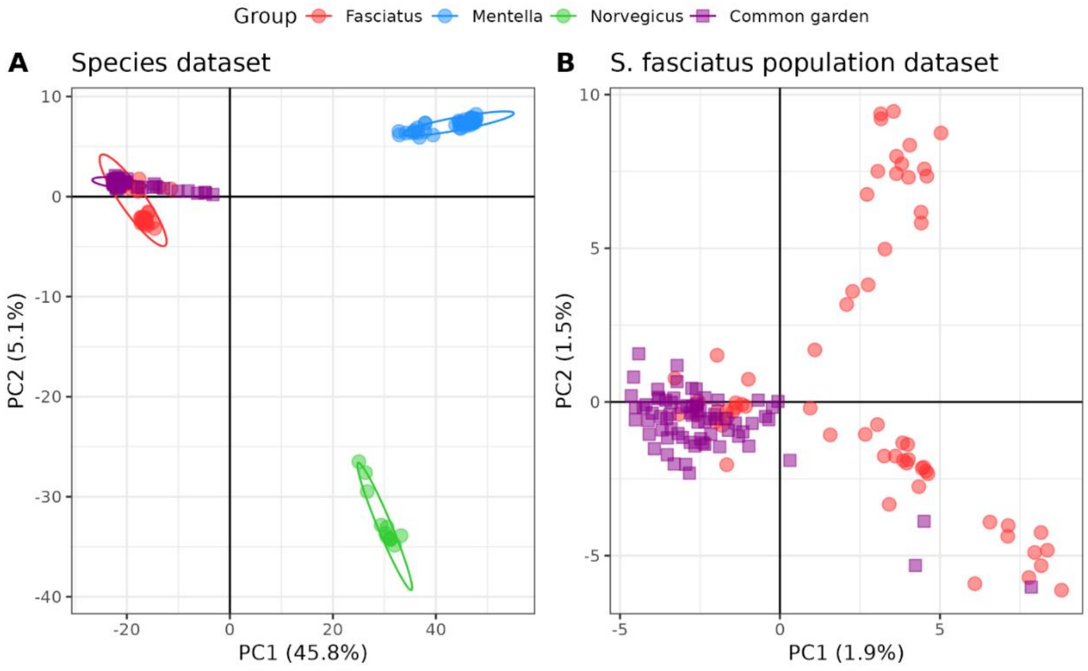

Fig. S2. Genetic variation of *S. fasciatus*. Principal component analysis of genetic variation observed at the individual level for A) the species dataset and B) the population dataset. Common garden refers to the tank experiment.

Journal of Experimental Biology • Supplementary information

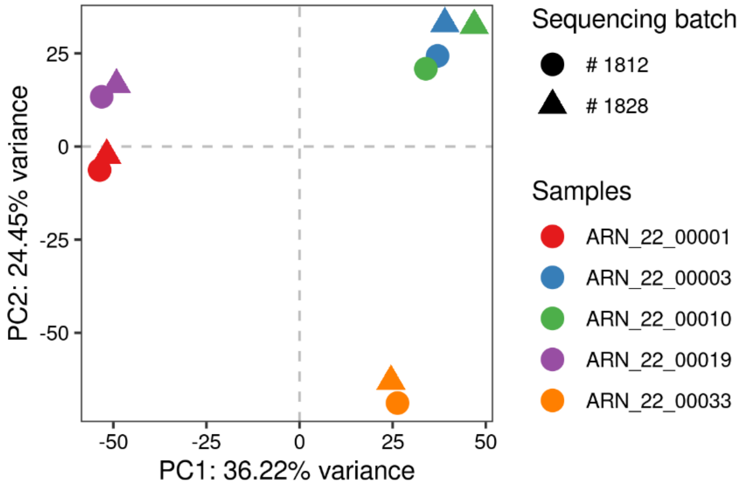

Fig. S3. Sequencing batch effect on measured transcript expression level. Principal component analysis of transcript expression level for samples (colors) sequenced through different sequencing batches (shapes). Redundancy analysis revealed that sequencing batch as no effect on measured transcript expression level (*adjusted* R^2 = 3,66 %; P = 0.131). Only five up to the six samples sequenced in both sequencing batches are illustrated, given that one of them displayed an excess of rRNA (see Results section).

Journal of Experimental Biology • Supplementary information

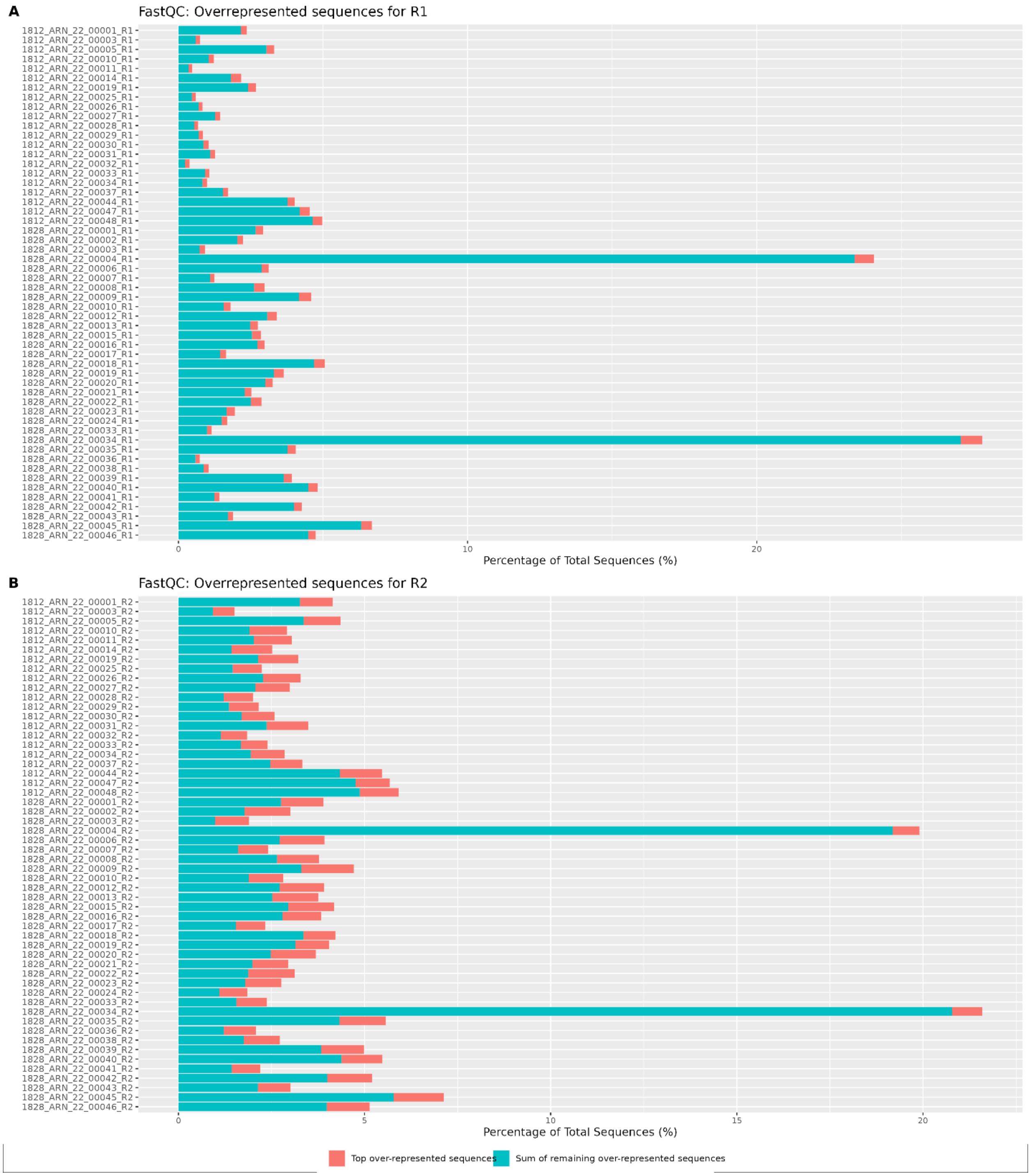

Fig. S4. FastQC and MultiQC output of overrepresented sequences. R1 (A) and R2 (B) sequence quality check were performed on trimmed reads, after TrimGalore! Steps.

Journal of Experimental Biology • Supplementary information

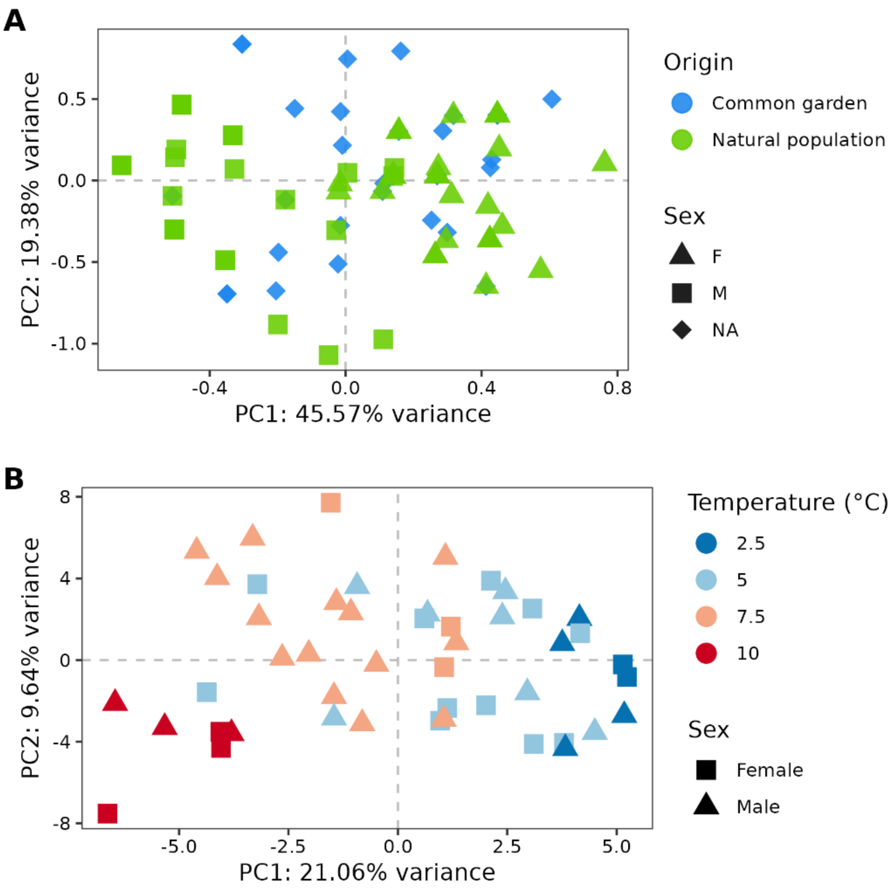

Fig. S5. Sex effect on gene expression. A. Genetic differentiation between male and female. Principal component analysis (PCA) on six sex-specific SNPs for individuals from natural environments (green, Natural population) with known sex and used to identify sex (shape, square for female and triangle for male) of individuals from the basin experiment (blue, Common garden). Genetic data and samples from natural environments came from a distinct study using dd-RAD sequencing (Supplementary Materials and Methods). Briefly, sex visual identification were performed on adult and mature individuals (lengths > 220 mmm). AssignPOP (Chen et al. 2018) was used to identify sex-linked SNPs and to assign sex of common garden individuals. **B. Sex effect on gene expression.** PCA on transcript expression level of different sex (shape) acclimated in four temperature (color), for individuals used for the RNA-seq analysis.

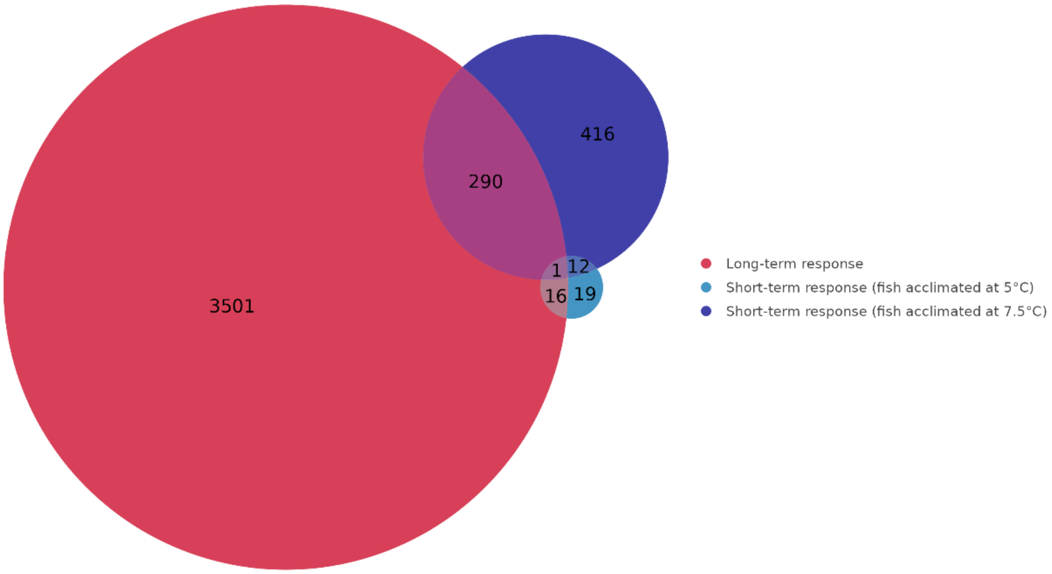

Fig. S6. Differentially expressed transcripts (DETs) among all laboratory experiments. Venn diagram representing the number of DETs in response to long and short-term temperature stress exposure.

Journal of Experimental Biology • Supplementary information

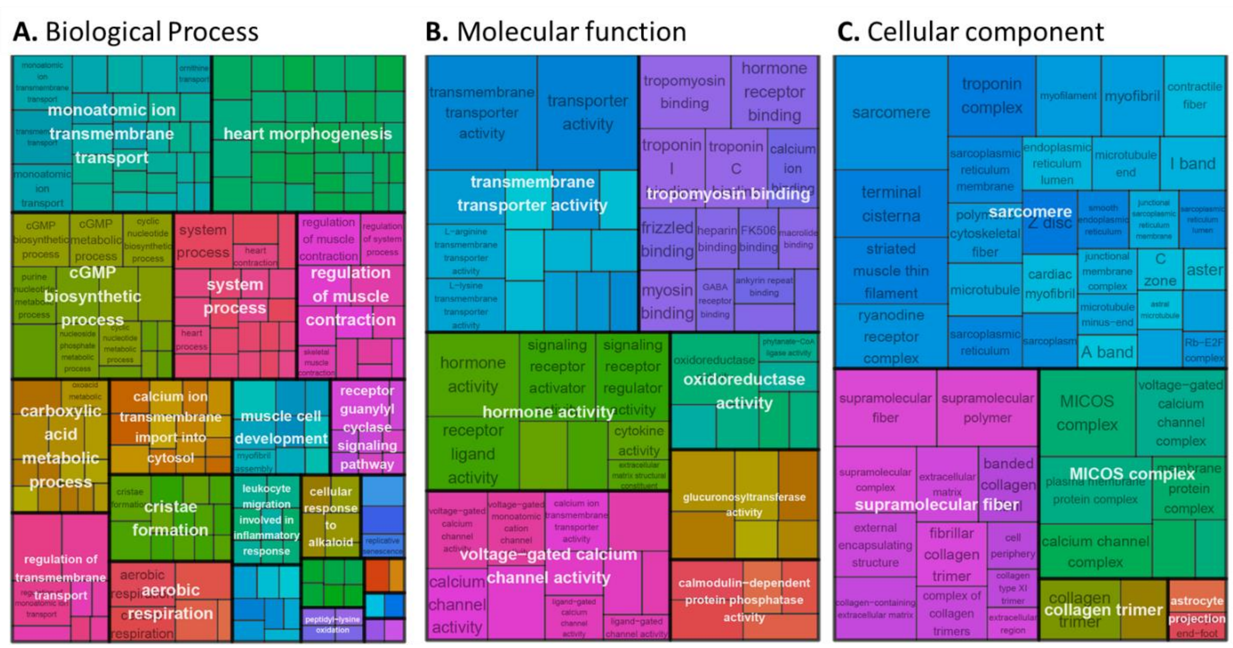

Fig. S7. GO terms associated to group L01 of DETs in the long-term response to temperature change. Group L01 DETs is characterized by decreasing gene expression level when temperature increased (see Fig. 1C). Treemap for (A) biological process, (B) molecular function and (C) cellular component where GO terms were grouped (color) based on their semantic similarity, and the space used by the term is proportional to the -log10(adjusted *P-value*), hence the gene function candidate probability.

Journal of Experimental Biology • Supplementary information

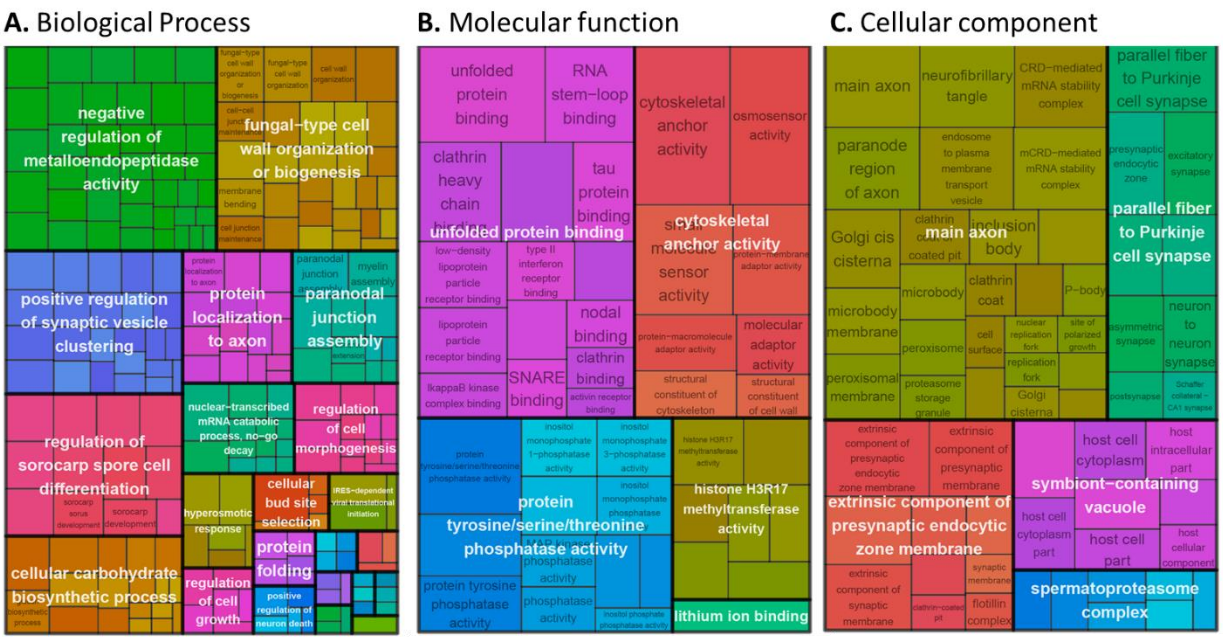

Fig. S8. GO terms associated to group L02 of DETs in the long-term response to temperature change. Group L02 DETs is characterized by increasing gene expression level when temperature increased (see Fig. 1C). Treemap for (A) biological process, (B) molecular function and (C) cellular component where GO terms were grouped (color) based on their semantic similarity, and the space used by the term is proportional to the -log10(adjusted *P-value*), hence the gene function candidate probability.

Journal of Experimental Biology • Supplementary information

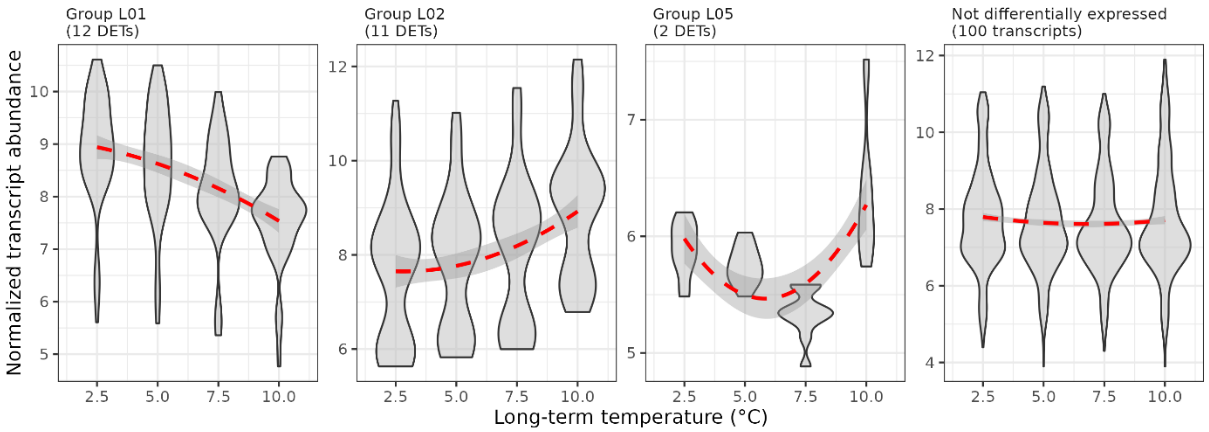

Fig. S9. Expression level of transcript associated to heat-shock proteins (HSPs). Normalized transcript abundance were obtained using the variance stabilizing transformation as implemented in DESeq2. Transcripts associated to HSPs and detected to be differentially expressed (DETs) among temperatures were grouped according to the expression moduled they belong to, including transcripts that were down reglulated (Group L01) or upregulated at higher temperatures (Group L02 and L05).

Table S1. Comparison of redundancy analysis (RDA) models assessing the effect of temperature treatment on fish gene expression, with and without sex as a covariate. Models include different combinations of explanatory variables: long-term temperature, exposure duration, sex (genetically identified; see Fig. S5), and short-term temperature (temperature during the final 24 h of the experiment). The levels of each categorical variable are specified. Adjusted R^2 values indicate the proportion of variance in transcript count uniquely explained by each factor, as estimated using partial RDA. Associated P-values indicate statistical significance based on 999 permutations. Interaction terms are denoted by "×".

Experiment	RDA models	Effects	Levels	adjusted R^2	P-value
Long-term temperature exposure	Transcript normalized count ~ Long-term temperature + Exposure duration	Long-term temperature	2.5 °C, 5.0 °C, 7.5 °C or 10.0 °C	22.67%	0.001
		Exposure duration	10 and 3 months	1.05%	0.102
	Transcript normalized count ~ Long-term temperature + Exposure duration + Sex	Long-term temperature	2.5 °C, 5.0 °C, 7.5 °C or 10.0 °C	21.68%	0.001
		Exposure duration	10 and 3 months	1.08%	0.287
		Sex	Male or Female	0.00%	0.679
Short-term temperature exposure	Transcript normalized count ~ Long-term temperature * Short-term temperature	Long-term temperature	5.0 °C or 7.5 °C	3.43%	0.005
		Short-term temperature	2.5 °C, 5.0 °C, 7.5 °C or 10.0 °C	2.49%	0.008
		Long-term × Short-term interaction		2.49%	0.020
	Transcript normalized count ~ Long-term temperature * Short-term temperature + Sex	Long-term temperature	5.0 °C or 7.5 °C	3.34%	0.007
		Short-term temperature	2.5 °C, 5.0 °C, 7.5 °C or 10.0 °C	2.56%	0.013
		Long-term × Short-term interaction		2.55%	0.021
		Sex	Male or Female	0.00%	0.879

References

Andrews, S. 2010. FastQC: A quality control tool for high throughput sequence data. Available from https://www.bioinformatics.babraham.ac.uk/projects/fastqc/.

Bolger, A.M., Lohse, M., and Usadel, B. 2014. Trimmomatic: A flexible trimmer for Illumina sequence data. Bioinformatics **30**(15): 2114–2120. doi:10.1093/bioinformatics/btu170.

Bourret, A., Benoit, H.P., Senay, C., and Parent, G.J. In preparation. Ubiquitous depth-related population structure within two sympatric Sebastes species and unequal contribution to massive recruitment events in the Northwest Atlantic.

Catchen, J., Hohenlohe, P.A., Bassham, S., Amores, A., and Cresko, W.A. 2013. Stacks: an analysis tool set for population genomics. Mol. Ecol. **22**(11): 3124–3140. doi:10.1111/mec.12354.

Chen K-Y, Marschall EA, Sovic MG, Fries AC, Gibbs HL, Ludsin SA. assignPOP: An r package for population assignment using genetic, non-genetic, or integrated data in a machine-learning framework. Methods Ecol Evol. 2018; 9: 439–446. doi.org/10.1111/2041-210X.12897

Ewels, P., Magnusson, M., Lundin, S., and Käller, M. 2016. MultiQC: Summarize analysis results for multiple tools and samples in a single report. Bioinformatics **32**(19): 3047–3048. doi:10.1093/bioinformatics/btw354.

Jombart, T., Devillard, S., Dufour, A.B., and Pontier, D. 2008. Revealing cryptic spatial patterns in genetic variability by a new multivariate method. Heredity (Edinb). **101**(1): 92–103. doi:10.1038/hdy.2008.34.

Li, H., and Durbin, R. 2009. Fast and accurate short read alignment with Burrows-Wheeler transform. Bioinformatics **25**(14): 1754–1760. doi:10.1093/bioinformatics/btp324.

Poland, J.A., Brown, P.J., Sorrells, M.E., and Jannink, J.L. 2012. Development of high-density genetic maps for barley and wheat using a novel two-enzyme genotyping-by-sequencing approach. PLoS One **7**(2). doi:10.1371/journal.pone.0032253.
